# Metachronous rectal metastasis from pulmonary adenocarcinoma after 11 years of chemo-, immuno-, and radiotherapy for recurrent lesions: a case report

**DOI:** 10.1186/s40792-019-0722-6

**Published:** 2019-10-24

**Authors:** Yozo Suzuki, Mitsunobu Imasato, Yujiro Nakahara, Atsushi Naito, Manabu Mikamori, Masahisa Ohtsuka, Kenta Furukawa, Jeong Ho Moon, Tadafumi Asaoka, Kentaro Kishi, Hironao Yasuoka, Kiyoshi Komuta, Hiroki Akamatsu

**Affiliations:** 1 Department of Gastroenterological Surgery, Osaka Police Hospital, Tennoji-Ku Kitayamacho 10-31, Osaka City, Osaka 543-0035 Japan; 2 Department of Pathology, Osaka Police Hospital, Tennoji-Ku Kitayamacho 10-31, Osaka City, Osaka 543-0035 Japan; 3 Department of Respiratory Medicine, Osaka Police Hospital, Tennoji-Ku Kitayamacho 10-31, Osaka City, Osaka 543-0035 Japan; 4 Department of Respiratory Medicine, Daini Osaka Police Hospital, Tennoji-Ku Karasugatsuji 2-6-40, Osaka City, Osaka 543-8922 Japan

**Keywords:** Rectal metastasis, Pulmonary adenocarcinoma, Palliative surgery, Chemotherapy, Immunotherapy

## Abstract

**Background:**

Rectal metastasis from pulmonary adenocarcinoma is rare, and it has been regarded as an end-stage phenomenon. Recently, however, advances in lung cancer treatment have improved the chance of long-term survival of patients with unresectable distant metastases. We describe the occurrence and management of metastatic spread of a pulmonary carcinoma to the rectum.

**Case presentation:**

The patient was a 79-year-old woman who had undergone thoracoscopic left lobectomy for pulmonary adenocarcinoma and then, over the next 11 years, various drugs (carboplatin + paclitaxel (as adjuvant therapy), gefitinib, gemcitabine + vinorelbine, S1 (an oral 5-fluorouracil-based drug), carboplatin + pemetrexed + bevacizumab, erlotinib, nivolumab, afatinib, and carboplatin+ S1) were administered, especially for hilar and mediastinal lymph node recurrences. During the eleventh postoperative year, left and right iliac bone metastases were detected, and radiation therapy was undertaken for local control of these lesions. When ^18^F-fluorodeoxyglucose positron emission tomography was performed for evaluation of the disease, tracer accumulation in the upper rectum was seen. Colonoscopic examination of the rectum revealed an intramural mass with central ulceration, and the mass was diagnosed histologically as an adenocarcinoma. The bone metastases appeared to be controlled, and the patient’s performance status was good, but she had suffered constipation for about a year and desired treatment. Thus, laparoscopic low anterior resection was performed. Histopathologic analysis revealed a moderately differentiated adenocarcinoma existing mainly between the submucosa and serosa, and immunohistochemical analysis showed the tumor to be positive for cytokeratin (CK) 7, negative for CK20, positive for thyroid transcription factor-1, and negative for special AT-rich sequence-binding protein 2 and caudal type homeobox 2, confirming the diagnosis of rectal metastasis from the primary pulmonary adenocarcinoma. The patient recovered well without any change in her functional status. Systemic chemotherapy was resumed, and she continues to do well, now 6 months after surgery.

**Conclusions:**

Surgery may be a good option for the management of an isolated rectal metastasis from pulmonary cancer in patients whose functional status is good.

## Background

Lung carcinoma is the leading cause of cancer-related death worldwide, and non-small cell lung cancer (NSCLC) accounts for the majority of cases. Metastatic lesions are found in more than half of patients at the time NSCLS is diagnosed, with the metastasis occurring most commonly in the lymph nodes, brain, liver, bone, or adrenal glands [1]. Gastrointestinal (GI) metastasis is found at autopsy in as many as 11.9% of cases [2], but clinically, GI metastasis is detected in only 0.19 to 0.33% of cases [3, 4], and metastatic spread to the rectum is rare.

## Case presentation

**Fig. 1:**
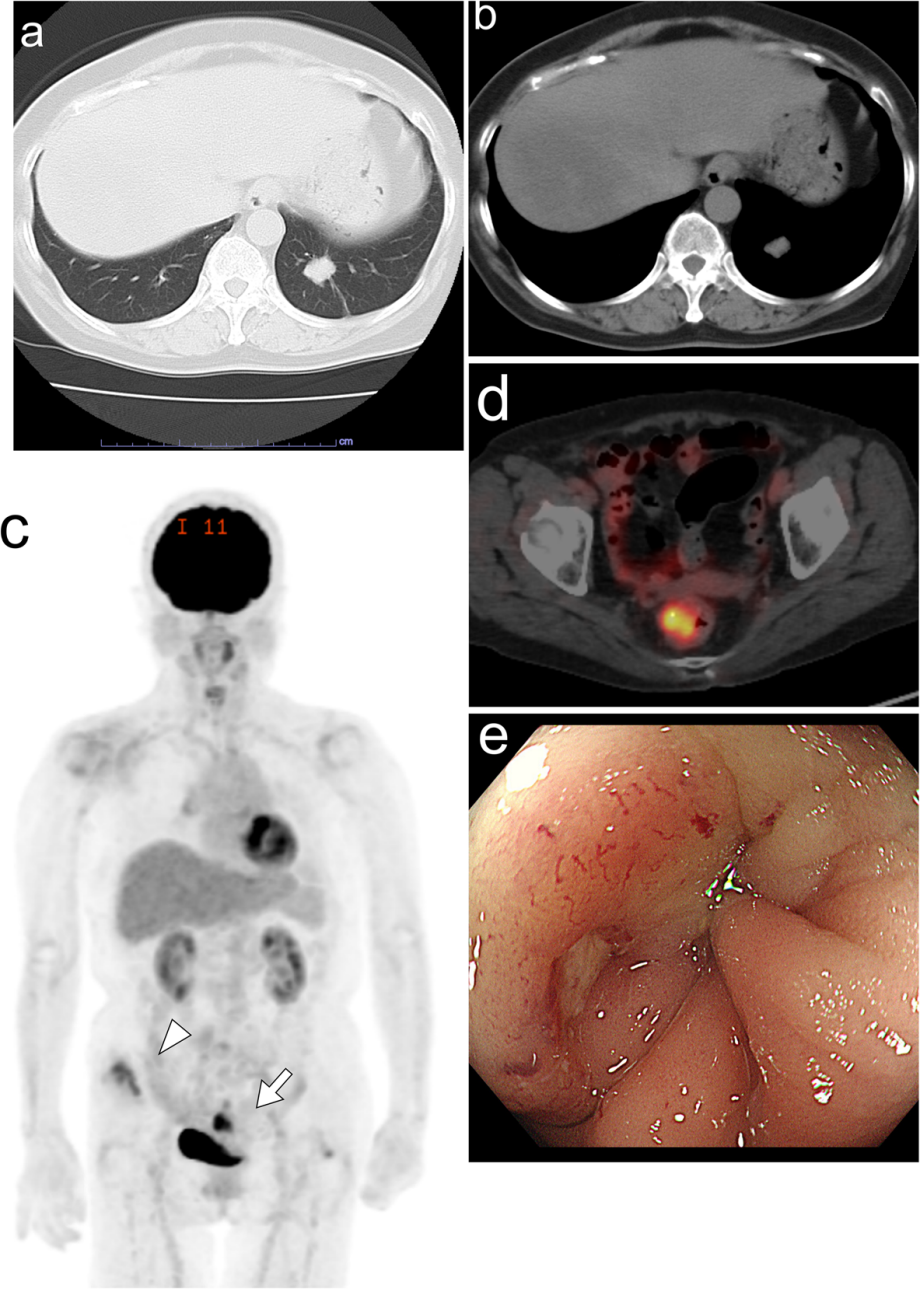
Images obtained over the clinical course. Chest computed tomography leading up to the initial diagnosis of primary pulmonary adenocarcinoma revealed a 2-cm nodule in the left lower lung lobe, as seen in the **a** lung window image and **b** mediastinal window image. Positron emission tomography leading to the diagnosis of rectal metastasis showed an accumulation of ^18^F-fluorodeoxyglucose in the upper rectum (arrow) and right iliac bone (arrowhead), as seen in **c** the whole body image and **d** horizontal section. Colonoscopy revealed **e** an intramural rectal mass with central ulceration

**Fig. 2:**
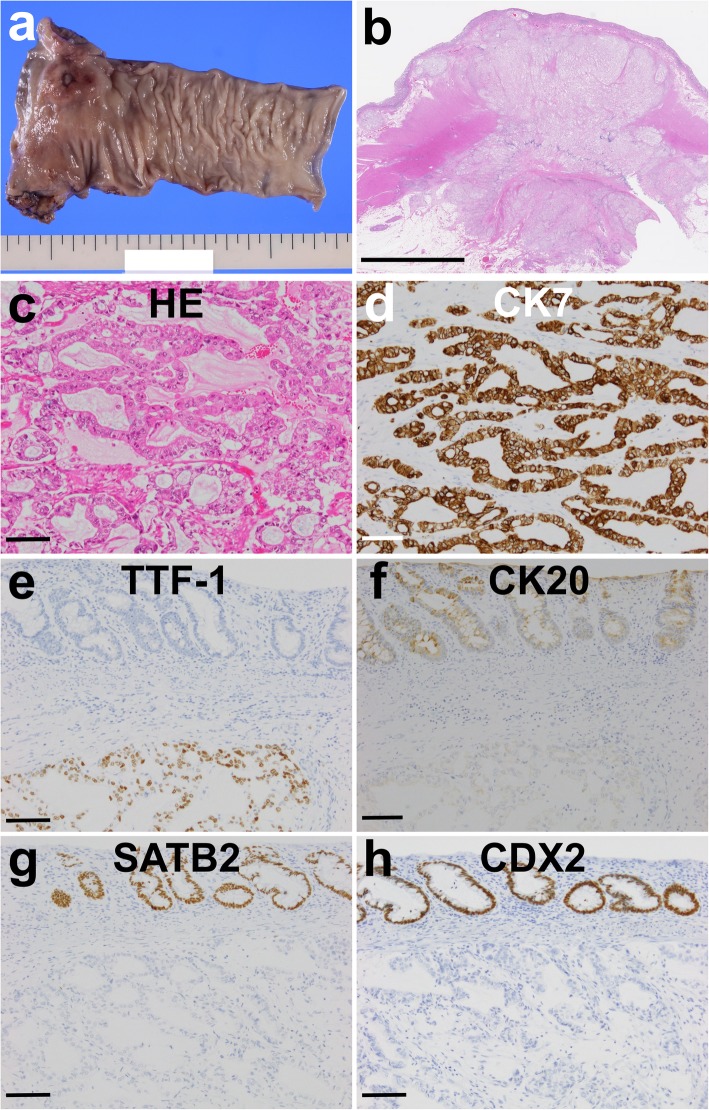
Histological and immunohistochemical (IHC) studies. Macroscopically, **a** the resected rectum specimen appeared to be a 30 × 25 mm tumor. A **b** loupe image of the tumor with hematoxylin and eosin (H&E)-stained section showed that the tumor was located mainly between the submucosal and subserosal layers. Histologic examination of this **c** and other H&E-stained sections showed that the tumor consisted of dysplastic cells with fused glandular structures and that it contained some mucinous components. Upon IHC staining, the tumor was positive for **d** anti-cytokeratin (CK) 7 and **e** thyroid transcription factor-1 (TTF-1), but negative for **f** CK20, **g** special AT-rich sequence-binding protein 2 (SATB2), and **h** anti-caudal type homeobox 2 (CDX2). IHC sections were stained with diaminobenzidine (brown) and counterstained with hematoxylin. Scale bars, 1 mm (**b**) and 100 μm (**c**–**h**). Original × 40 magnification (**c**–**h**)

A 79-year-old woman had undergone thoracoscopic left lobectomy for a well-differentiated acinar adenocarcinoma harboring an epidermal growth factor receptor gene mutation (pT1c pN2 cM0, stage IIIA [Union Internationale Contre le Cancer staging system]) (Fig. 1a, b) [5]. The metastatic node was found in the left pulmonary ligament, and lymphatic and vascular invasion were identified upon microscopic examination of the main tumor. Chemotherapy and immunotherapy were performed over a period of 11 years for hilar and mediastinal lymph node recurrences. Various drugs were used, and some of which were changed when apparent allergic reactions developed (4 courses of carboplatin + paclitaxel as an adjuvant therapy [6], gefitinib [7], gemcitabine + vinorelbine [8], S1 (an oral 5-fluorouracil-based drug) [9], carboplatin + pemetrexed + bevacizumab [10], erlotinib [11], nivolumab [12], afatinib [13], and carboplatin+ S1 [14]). The disease continued to progress, however, and radiation therapy had been undertaken for local control of left and right iliac bone metastases (40 Gy in 20 fractions and 50 Gy in 25 fractions, respectively). ^18^F-fluorodeoxyglucose positron emission tomography was performed for disease evaluation, and although the bone metastases had not progressed, accumulation (maximum standardized uptake value of 8.8) was seen in the upper rectum (Fig. 1c, d). Colonoscopy revealed an intramural mass with central ulceration in the rectum (Fig. 1e), and histologic analysis showed the tumor to be an adenocarcinoma. The bone metastases appeared to be controlled and the patient’s performance status [15] was 1, but she had suffered from constipation for about a year and desired treatment. Thus, we performed laparoscopic low anterior resection. Histopathologic analysis of the surgical specimen revealed moderately differentiated adenocarcinoma between submucosal and subserosal layers with a mucinous component and formation of dysplastic cells into fused glandular structures (Fig. 2a–c). The tumor cells were positive for cytokeratin (CK) 7 and thyroid transcription factor-1, and negative for CK20, special AT-rich sequence-binding protein 2, and caudal type homeobox 2 (Fig. 2d–h), together indicating that the tumor was a metastatic lesion originating from the lung adenocarcinoma [16–19]. The patient recovered well without a change in her functional status. S1 chemotherapy was resumed, and the patient has remained well in the 6 months that have passed since the surgery.

## Discussion

Of all primary lung cancers, large cell carcinoma poses the highest risk of GI metastasis, and adenocarcinoma poses the lowest risk [2, 20]. Although 5 cases of rectal metastasis were reported in a review of 366 cases of GI metastasis from primary lung cancers [20], the 5 derived from either a small cell or squamous cell lung cancer. To the best of our knowledge, ours is the first case of rectal metastasis from a pulmonary adenocarcinoma to be reported in the English literature. By the time NSCLC is diagnosed, the disease has, in most cases, reached an advanced stage [1]. GI metastasis from NSCLC is accompanied by extra-GI metastases in 70% of patients, and in most reported cases, the GI metastasis occurred within 1 year after diagnosis of the primary tumor [21]. The time interval between diagnosis of the primary tumor and detection of GI metastasis was exceptionally long in our case. The numerous chemo- and immunotherapy regimens that were applied might explain the long interval in our case; most of the therapies had controlled the disease for a half year or more. Recent advances in treatment have improved the survival of patients with NSCLC [22], and the chance of relatively long survival for patients with distant metastatic lesions that cannot be curatively resected has increased [21]. The prognosis of NSCLC with GI metastasis is typically poor; median overall survival time is approximately 3 months; and advanced age, extra-GI metastasis, and GI perforation are associated with a poor prognosis [3, 20, 23]. Few long-term survivors exist, and outcomes are better for patients in whom GI metastasis from NSCLC is diagnosed preoperatively [23]. Our patient was of advanced age, and extra-GI metastasis had occurred, but there was no rectal perforation and, notably, the extra-GI metastasis was controlled. Surgical management turned out to be a good option for her.

Our experience in this case of metachronous rectal metastasis from pulmonary adenocarcinoma after 11 years of chemotherapy, immunotherapy, and radiotherapy for recurrent lesions suggests that surgical management is a reasonable option for patients whose disease is controlled.

## Conclusion

We believe that clinicians should take surgical intervention into consideration for the management of rectal metastases from NSCLC in patients whose disease is controlled and whose performance status is good.

## Data Availability

The datasets supporting the conclusions of this article are included within the article and its additional files.

## Abbreviations

CDX2: Caudal type homeobox 2
CK: Cytokeratin
GI: Gastrointestinal
H&E: Hematoxylin and eosin
IHC: Immunohistochemical
NSCLC: Non-small cell lung cancer
SATB2: Special AT-rich sequence-binding protein 2
TTF-1: Thyroid transcription factor-1
